# Gadolinium retention and clearance after administration of macrocyclic magnetic resonance contrast agents to rats

**DOI:** 10.1007/s00247-019-04439-9

**Published:** 2019-06-28

**Authors:** Simona Bussi, Federico Maisano, Fabio Tedoldi, Miles A. Kirchin

**Affiliations:** Bracco Imaging SpA, Via Caduti Di Marcinelle, 20134 Milan, Italy

Dear Editors,

We read with interest the article by Dr. Blumfield et al. [1] in the March 2019 issue of *Pediatric Radiology* on MR signal intensity changes and tissue gadolinium (Gd) retention, with emphasis on pediatric patients. On page 454 the authors reported the results of a study by Bussi et al. [2], making the following statement: “*A recent study by Bussi et al. [68] compared the three macrocyclic agents Dotarem, ProHance and Gadovist in rats that were sacrificed 28 days following multiple administrations of one of these GBCAs. The authors found significantly lower concentrations of gadolinium in rats injected with Dotarem when compared to the other two agents, in the cerebrum, cerebellum, femur and renal tissues*.” Later, on the same page, the authors stated the following: “*Furthermore there are differences between macrocyclic agents that are related to their chemical stability, with a higher clearance rate of Dotarem, which is ionic and hence more stable than ProHance and Gadovist*.” These statements are factually incorrect.

In the study in question, Bussi et al. [2] made 20 repeated administrations of ProHance (gadoteridol; Bracco Diagnostics Inc., Milan, Italy), Dotarem (gadoterate meglumine; Guerbet LLC, Villepinte, France) or Gadovist (gadobutrol; Bayer, Leverkusen, Germany) to rats (15/group) and then determined gadolinium levels in the cerebrum, cerebellum, liver, kidneys, skin and blood by inductively coupled plasma-mass spectrometry (ICP-MS) after a 28-day recovery period. Contrary to the statement of Dr. Blumfield et al. [1], Bussi et al. showed significantly lower levels of gadolinium in all soft-tissue organs after the cumulative administration of ProHance than after Dotarem or Gadovist: 0.150±0.022 vs. 0.292±0.057 and 0.287±0.056 nmol/g, respectively (*P*<0.001), in the cerebellum; 0.116±0.036 vs. 0.250±0.032 and 0.263±0.045 nmol/g, respectively (*P*<0.001), in the cerebrum; 25±13 vs. 139±88 (*P*<0.01) and 204±109 nmol/g (*P*<0.001), respectively, in the kidneys. Significantly (*P*<0.001) higher gadolinium levels were noted in the femur with Gadovist (8.60±2.04 nmol/g) compared to Dotarem (5.69±1.75 nmol/g) while the mean value for ProHance (7.48±1.37 nmol/g) was only marginally significantly higher than the mean value for Dotarem (*P*<0.05).

It is worth emphasizing that other authors have similarly found lower levels of gadolinium in rat brain and body tissues after administration of ProHance compared to Dotarem and Gadovist, particularly in the first days and weeks after administration, indicating a more rapid clearance of ProHance than Dotarem or Gadovist [3, 4]. The differential clearance appears to reflect differences in the specific molecular properties of the agents rather than any effect of stability [5]. If animal studies are to be taken as indicative of the situation in humans, as Dr. Blumfield et al. [1] implied, then it is worth noting the vast differences in time-scale between the two species, with 1 rat year corresponding to approximately 30 human years [6]. This means that gadolinium levels measured at 28 days after the last contrast administration in the study by Bussi et al. [2] would equate to almost 2.5 years in human terms. This might be particularly relevant for MRI of pediatric subjects.

In conclusion, Dr. Blumfield et al. [1] have misunderstood the results of the study by Bussi et al. [2] and need to correct and clarify their statements to avoid misinformation and misinterpretation.
